# The double-hit protocol induces HFpEF and impairs myocardial ubiquitin-proteasome system performance in FVB/N mice

**DOI:** 10.3389/fphys.2023.1208153

**Published:** 2023-06-08

**Authors:** Jose R. Lira, Andrew L. Guymon, Liuqing Yang, Jack O. Sternburg, Samiksha Giri, Xuejun Wang

**Affiliations:** Division of Basic Biomedical Sciences, University of South Dakota Sanford School of Medicine, Vermillion, SD, United States

**Keywords:** heart failure with preserved ejection fraction (HFpEF), high fat diet (HFD), Nω-nitro-L-arginine methyl-ester (L-NAME), ubiquitin-proteasome system (UPS), FVB/N mice

## Abstract

Heart failure with preserved ejection fraction (HFpEF) is a leading cause of death and disability, with its prevalence surpassing that of heart failure with reduced ejection fraction. Obesity and hypertension are often associated with HFpEF. HFpEF can be modeled through simultaneous metabolic and hypertensive stresses in male C57BL/6N mice provoked by a combination treatment of a high-fat diet (HFD) and constitutive nitric oxide synthase inhibition by Nω-nitro-L-arginine methyl-ester (L-NAME). Ubiquitin-proteasome system (UPS) dysfunction was detected in many forms of cardiomyopathy, but whether it occurs in HFpEF remains unknown. We report successful modeling of HFpEF in male FVB/N mice and, by taking advantage of a transgenic UPS reporter mouse, we have detected myocardial UPS functioning impairment during HFpEF, suggesting a pathogenic role for impaired protein degradation in the development and progression of HFpEF.

## Introduction

Heart failure with preserved ejection fraction (HFpEF) is a lethal clinical syndrome associated with poor quality of life and substantial healthcare resource utilization comprising over half of all heart failure but with very limited pharmaceutical interventions (Dunlay et al., 2017). From clinical understanding of HFpEF in humans, HFpEF is strongly associated with numerous comorbidities including hypertension, obesity, diabetes, exercise intolerance, atrial fibrillation, chronic kidney disease, and coronary artery disease (Shah et al., 2013a). Notably, HFpEF is not strongly associated with acute coronary syndrome; instead, it is accompanied by a plethora of comparatively more chronic diseases. Such accompaniment substantially increases the difficulty of engineering a HFpEF mouse model that accurately recapitulates the disease in an efficient amount of time. In lieu of this, the “two-hit” HFpEF mouse model utilizes the two most dominant avenues of HFpEF pathology: hypertension and obesity/metabolic syndrome. The “two-hit” hypothesis subjected C57BL/6N wild-type mice to a treatment of high fat diet (HFD) and Nω-nitro-L-arginine methyl-ester (L-NAME) to produce a HFpEF mouse model that recapitulates numerous hallmarks of HFpEF including hypertension, obesity, left ventricular myocardial remodeling, exercise intolerance in the absence of significant histopathological, molecular, or strength abnormalities in skeletal muscle, increased LV filling pressure, increase in lung weight, cardiomyocyte hypertrophy, cardiac fibrosis, myocardial capillary rarefaction but maintained left ventricular (LV) ejection fraction (EF) (Schiattarella et al., 2019). The “two-hit” HFpEF mouse model has quickly become one of the leading HFpEF animal models.

The pathophysiology of HFpEF is complex and is not well understood. However, some hallmark characteristics remain. End-diastolic pressure elevation occurs due to an intricate interplay between diastolic malfunction, elusive systolic malfunction, and reduced atrial, LV, and arterial compliance (Gevaert et al., 2019). Most HFpEF patients demonstrate multiple pathological derailments, including cardiac and noncardiac dysfunction. Cardiac elements include diastolic dysfunction, reduced cardiac output reserve, atrial fibrillation, and coronary artery disease. In contrast, noncardiac elements include reduced vasodilation, increased arterial stiffness, ventilatory dysfunction, skeletal myopathy, activation of the autonomic nervous system, and renal dysfunction (Gevaert et al., 2019). Underlying pathologies may also be classified according to their contribution to endothelial damage (increased inflammation and decreased repair), exercise intolerance due to cardiac and noncardiac mechanisms, and comorbidities such as aging, metabolic syndrome, and iron deficiency (Gevaert et al., 2019). Clearly, numerous interactions account for the HFpEF phenotype, albeit some underlying pathologies may dominate in individual patients (Borlaug, 2014). Markedly important is the ambitious attempt to deepen our understanding of HFpEF by developing animal models that recapitulate not only the presenting symptoms of HFpEF but also the diverse array of molecular abnormalities.

The molecular pathophysiology of HFpEF is aberrantly complex and fluid, citing endothelial dysfunction in the setting of metabolic inflammation, abnormalities in nitric oxide (NO) synthesis and bioavailability (Chistiakov et al., 2015; Schiattarella et al., 2019), impeded endothelial repair (Gevaert et al., 2019), cardiomyocyte maladaptation impairing diastolic function (Leucker and Jones, 2014), fibroblast dysfunction, and recent discoveries of misfolded protein accumulation (Gonzalez-Lopez et al., 2015; Wang and Wang, 2020). Targeted degradation of misfolded proteins by the ubiquitin-proteasome system (UPS) is pivotal to protein quality control (Wang and Wang, 2020), a vital part of the mechanisms maintaining proteostasis in the cell (Frankowska et al., 2022). Approximately 13% of HFpEF patients suffer from wild-type transthyretin (TTR) amyloidosis stemming from increased cardiac protein deposition (Gonzalez-Lopez et al., 2015). Myocardial UPS functional insufficiency was observed in a widely used mouse model of cardiac proteinopathy where pre-amyloid oligomers are increased in cardiomyocytes (Chen et al., 2005; Liu et al., 2006). Animal model and cell culture studies have established both the sufficiency and, in some cases, the necessity of proteasome functional insufficiency as a significant pathogenic factor in the heart (Wang and Robbins, 2014). Clinically, the proteasome inhibiting drugs bortezomib and carfilzomib have been shown to cause cardiotoxicity, including heart failure (Enrico et al., 2007; Das et al., 2022; Makris et al., 2022; Georgiopoulos et al., 2023).

Potential treatments addressing cardiac proteotoxicity seem within reach, further emphasizing the urgency of exploring impaired proteostasis as a pathological basis of HFpEF. Overexpression of proteasome activator 28α (PA28α) is cardioprotective against numerous challenges without altering normal protein turnover or cardiac function (Li et al., 2011; Wang and Wang, 2020). Potentially meeting HFpEF at the intersection of diabetes and HF, overexpression of PA28α attenuates diabetes-induced proteotoxic stress and cardiomyopathy (Li et al., 2017). An upregulated unfolded protein response (UPR) ameliorated the diastolic dysfunction essential to HFpEF, suggesting that impaired proteostasis plays a key role in HFpEF pathogenesis (Schiattarella et al., 2019). By activating the cGMP-dependent protein kinase (PKG), phosphodiesterase 5 (PDE5) inhibition by sildenafil slows down cardiac disease progression in a mouse model of CryAB^R120G^-based cardiac proteinopathy (Ranek et al., 2013); and inhibition of PDE1 induces cAMP-dependent protein kinase (PKA) and PKG-mediated promotion of proteasomal degradation of misfolded proteins and thereby effectively treats diastolic malfunction and delays premature death in CryAB^R120G^-based cardiac proteinopathy mice (Zhang et al., 2019).

Impaired myocardial UPS performance has been observed in both pressure overload induced HF and diabetic cardiomyopathy (Ranek et al., 2015; Li et al., 2017). Plus, both myocardial UPS impairment and diastolic malfunction are co-existed in mouse models of cardiac proteinopathy (Zhang et al., 2019). Since metabolic syndrome/diabetes and hypertension are common co-morbidities of HFpEF, it is very likely that myocardial UPS functioning is impaired during HFpEF but no reported studies have examined that. Perhaps the most powerful tool to probe *in vivo* UPS performance is the transgenic mouse model with ubiquitous expression of a green fluorescence protein (GFP) that has been modified with carboxyl fusion of degron CL-1 (Bence et al., 2001), known as GFPdgn that has been validated as an inverse UPS functioning reporter (Kumarapeli et al., 2005). Employing the GFPdgn mice has allowed researchers to unveil *in vivo* UPS dysfunction in various cardiac disorders (Chen et al., 2005; Liu et al., 2006; Li et al., 2011; Li et al., 2017)*.* Applying the “two-hit” hypothesis to FVB/N GFPdgn transgenic mice may provide an animal model of HFpEF that accurately recapitulates the disease’s diverse etiologies and pathologies while probing into the disease’s proteotoxic nature and UPS dysfunction.

Here we report that the HFD+L-NAME treatment can induce HFpEF in FVB/N male mice; myocardial UPS functioning is impaired during HFpEF and warrants further comprehensive investigation.

## Methods

### Experimental animals and treatments

All experiments followed ethical and legal standards according to The Guide for the Care and Use of Laboratory Animals published by the US National Institutes of Health (NIH Publication 8^th^ edition, update 2011) and the Institutional Animal Care and Use Committee of the University of South Dakota. The creation and validation of the GFPdgn transgenic mice for monitoring the dynamic changes in UPS performance *in vivo* was previously described (Kumarapeli et al., 2005). Littermate male GFPdgn transgenic and non-transgenic mice in the FVB/N inbred background at 6–7 months of age were randomly assigned to the CHOW group or the HFD+L-NAME group throughout the study. The CHOW (control diet) group had unrestricted access to a standard murine chow diet of 19.1% protein, 6.5% fat, and 47% carbohydrates (2020X from Teklad) and water. The HFD+L-NAME group had unrestricted access to an HFD where 60% of the calories were from fat (lard and soybean oil), 20% from proteins, and 20% from carbohydrates (D12492, Research Diets, Inc) and water spiked with L-NAME (0.5 g/L; Sigma Aldrich, Catalog #: N5751-10G). Body weight of each mouse was measured weekly for 18 weeks at the same time of day. The age of mice at the initiation time of this study (6–7 months of age), an age that should be more clinically relevant to HFpEF that naturally occurs in humans compared to the much younger age (8–10 weeks of age) used in the prior report of this double-hit method (Schiattarella et al., 2019).

### Glucose tolerance test

A bolus intraperitoneal injection of 20% glucose solution (1 g/kg in water) was conducted after 6-h fasting. Blood from the tail was collected and measured immediately before (0 min) and 15, 30, 60, 90, and 120 min after the injection using Bayer Contour^®^ Next EZ Glucose Meter Kit and Contour^®^ Next Blood Glucose Test Strips.

### Exercise tolerance test

Mice ran on an Exer-3/6 treadmill with Stimulus Detection (Columbus Instruments, Columbus, OH) on a 10-degree incline. The day before the test, animals were subjected to an acclimation at 5 m/min for 4 min, then increased to 10 m/min for 10 min. For the final test, mice were randomly grouped in a lane and perform at 5 m/min for 4 min then at 14 m/min for 2 min. Speed was increased by 2 m/min every 2 min until exhaustion. Exhaustion is defined as the animal getting shocked continuously for 5 s. The Treadmill Software (Columbus Instruments, Columbus, OH) measured running distance, time ran, and exhaustion.

### Non-invasive blood pressure measurement

The tail-cuff method was used to measure systolic blood pressure. Animals were trained in the instrument for at least 2 months before the final test. The mice were acclimated in the temperature-controlled chamber for 15 min, and results were measured, then another 15 min were allowed for resting data. Blood pressure was then recorded for 4 days, and readings averaged.

### Echocardiography

Echocardiography was performed as previously reported in a double-blinded manner (Zhang et al., 2019). In brief, mice were kept in light anesthesia with inhalation of Isoflurane (4% for induction and 1.5% for maintenance) via a face mask. Transthoracic echocardiography was performed using the Visual Sonics Vevo 3100 system and a 40-MHz probe (FUJIFILM Visual Sonics, Toronto, ON, Canada). A two-dimensional guided M-mode episode was acquired through the left ventricular (LV) anterior and posterior walls at short axis view. Parameters of LV were derived from primary measurements using Vevo LAB software.

### Western blot analysis

Ventricular myocardial tissues were homogenized in 1x loading buffer (41 mM tris-HCI, 1.2% SDS, 8% glycerol). The homogenates were boiled for 5 min, then centrifuged for 20 min at 4°C at 14,000 rpm, and the supernatant was collected. Protein concentration of the samples were measured using Pierce BCA Protein Assay Reagent (Thermofisher, Rockford, Illinois). Equal amounts of proteins were loaded to each lane of a 10% SDS-PAGE gel and fractionated by electrophoresis, then electrically transferred to a PVDF membrane overnight in 4°C. The PVDF membrane was washed and incubated in blocking buffer for 60 min before being probed with the primary antibody overnight followed by incubation with HRP-conjugated secondary antibodies. The bound secondary antibodies on the PVDF were then detected with the SuperSignal West Pico PLUS Chemiluminescent Substrates (ThermoScientific) and imaged with a ChemiDoc MP imaging system (Bio-Rad). Densitometry quantification was performed using the ImageLab software (Bio-Rad).

### RNA isolation, reverse transcription, and real time PCR (qPCR)

Total RNA was extracted from left ventricular myocardium using TRI Reagent^®^ (Molecular Research Center Inc., Cincinnati, OH). Thermo Scientific™ NanoDrop 2000 UV Spectrophotometer was used to determine the concentration and purity of the RNA. Reverse transcription utilized the High Capacity cDNA Reverse Transcription Kit (Applied Biosystems™) and a total of 900 ng of extracted RNA as template. qPCR reactions used 2 μL of 1:10 nuclease-free water diluted cDNA solution and PowerUp SYBR Green master mix (Applied Biosystems) for the following gene products: Atrial Natriuretic Factor (*Nppa*), Brain Natriuretic Peptide (*Nppb*), *GFPdgn*, Phospholamban (*Pln*), and Glyceraldehyde-3-phosphate dehydrogenase (*GAPDH*). All reactions for target genes (*Nppa*, *Nppb*, *GFPdgn*, and *Pln*) were performed in duplicate. The 2^−ΔΔCT^ relative quantification method using *GAPDH* as the normalization gene was used to compute the relative expression of the target genes. The primer sequences for the qPCR reactions are shown in Table 1.

**TABLE 1:** qPCR Primer Sequences.

mRNA target	Sense/Antisense	Primer sequence (5′-3′)
*Gapdh*	Sense	ATG​ACA​TCA​AGA​AGG​TGG​TG
	Antisense	CAT​ACC​AGG​AAA​TGA​GCT​TG
*Nppa*	Sense	GGA​GGA​GAA​GAT​GCC​GGT​AGA
	Antisense	GCT​TCC​TCA​GTC​TGC​TCA​CTC
*Nppb*	Sense	CTG​CTG​GAG​CTG​ATA​AGA​GA
	Antisense	TGC​CCA​AAG​CAG​CTT​GAG​AT
*GFPdgn*	Sense	TCT​ATA​TCA​TGG​CCG​ACA​AGC​AGA
	Antisense	ACT​GGG​TGC​TCA​GGT​AGT​GGT​TGT
*Pln*	Sense	CAA​TAC​CTC​ACT​CGC​TCG​GC
	Antisense	GCGGTGCGTTGCTTCCC

### Statistical methods

GraphPad Prism software 9.2.0 (GraphPad Software, Inc., La Jolla, CA) was used for all statistical analysis. Results are presented as the mean ± SEM. Datum was deemed an outlier according to the Grubbs test (*p* < 0.05). Two-tailed unpaired Student’s t-test was used unless indicated otherwise. In addition, the Welches correction for unequal variance was used when analyzing gene expression results. Researchers were blinded to the individual genotypes during the data collection and data analysis. A *p*-value or adjusted *p*-value of <0.05 was considered statistically significant.

## Results

### The “double-hit” method induced metabolic syndrome in FVB/N mice

**FIGURE 1:**
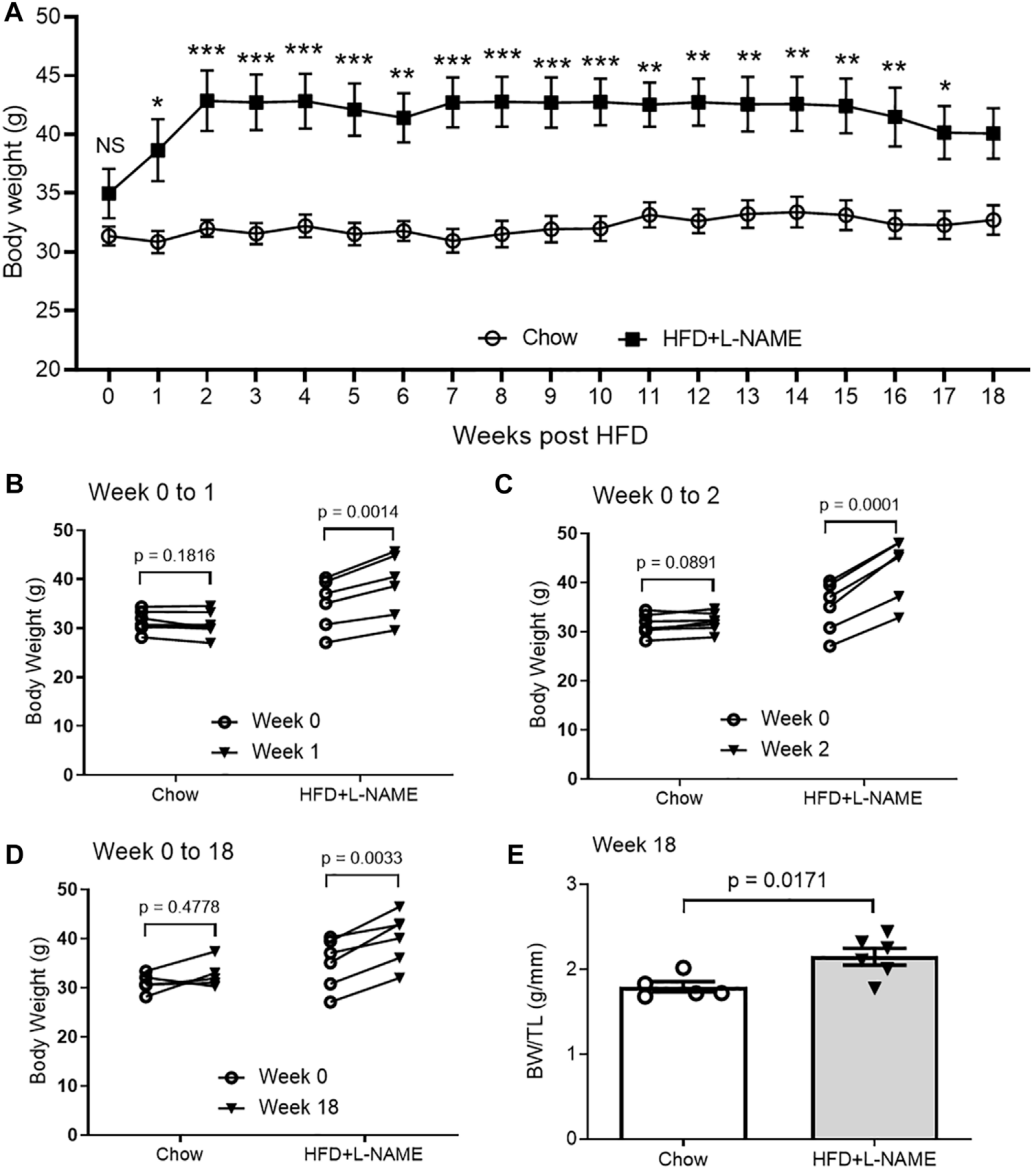
Changes in body weight (BW) of HFD+L-NAME or chow treated mice. **(A)**, Comparison of the time courses of changes in BW measured immediately before (Week 0) and weekly after the initiation of the HFD or control diet (Chow) treatment. Shown are line graphs superimposed by mean ± SEM of each time point. The *p* values shown are adjusted *p* values derived from multiple two-sided unpaired *t*-tests with correction for multiple comparisons using the Bonferroni-Dunn method; NS, not significant; *p < 0.05, **p < 0.01, ***p < 0.001. The Chow group started with 7 mice but a mouse dropped out at Week 8 and another at Week 14. The HFD+L-NAME group had 6 mice throughout. **(B–D)**, Longitudinal comparison of BW from Week 0 to Week 1 **(B)**, to Week 2 **(C)**, or to Week 18 **(D)**. Each circle represents a mouse. *P* values are derived from two-sided paired *t*-tests. **(E)**, BW/TL ratio measured at terminal experiment (Week 18). TL, tibial length. Shown are scatter plots superimposed by mean ± SEM; each circle or dot represents a mouse. The *p*-value is derived from two-sided unpaired *t*-test.

HFD can induce obesity and the latter inevitably increases body weight (BW). Thus, we measured mouse BW immediately before (week 0) and weekly after initiation of the HFD+L-NAME treatment. No significant changes in BW were observed in the Chow group throughout the 18 weeks of study (Figure 1). The difference in BW between the Chow and the HFD+L-NAME groups was not statistically significant at week 0 (*p* > 0.9999) but became significant from week 1 to week 17 (Figure 1A). Self-comparisons show significant BW increases in the HFD+L-NAME group as early as week 1 (*p* = 0.0014; Figure 1B) and the increases peaked at week 2 (*p* = 0.0001; Figures 1A,C) and plateaued thereafter (Figures 1A–D). Longitudinal BW comparisons between week 0 and week 1 (Figure 1B, *p* = 0.182 and 0.001 for the Chow and the HFD+L-NAME groups, respectively), between week 0 and week 2 (Figure 1C, *p* = 0.0891 and 0.0001), or between week 0 and week 18 (Figure 1D, *p* = 0.4778 and 0.0033) are also summarized in Figure 1. The BW to tibial length (TL) ratio (BW/TL) of the HFD+L-NAME group was greater than that of the Chow group (*p* = 0.0171, Figure 1E) at week 18 when the terminal experiments were performed.

**FIGURE 2:**
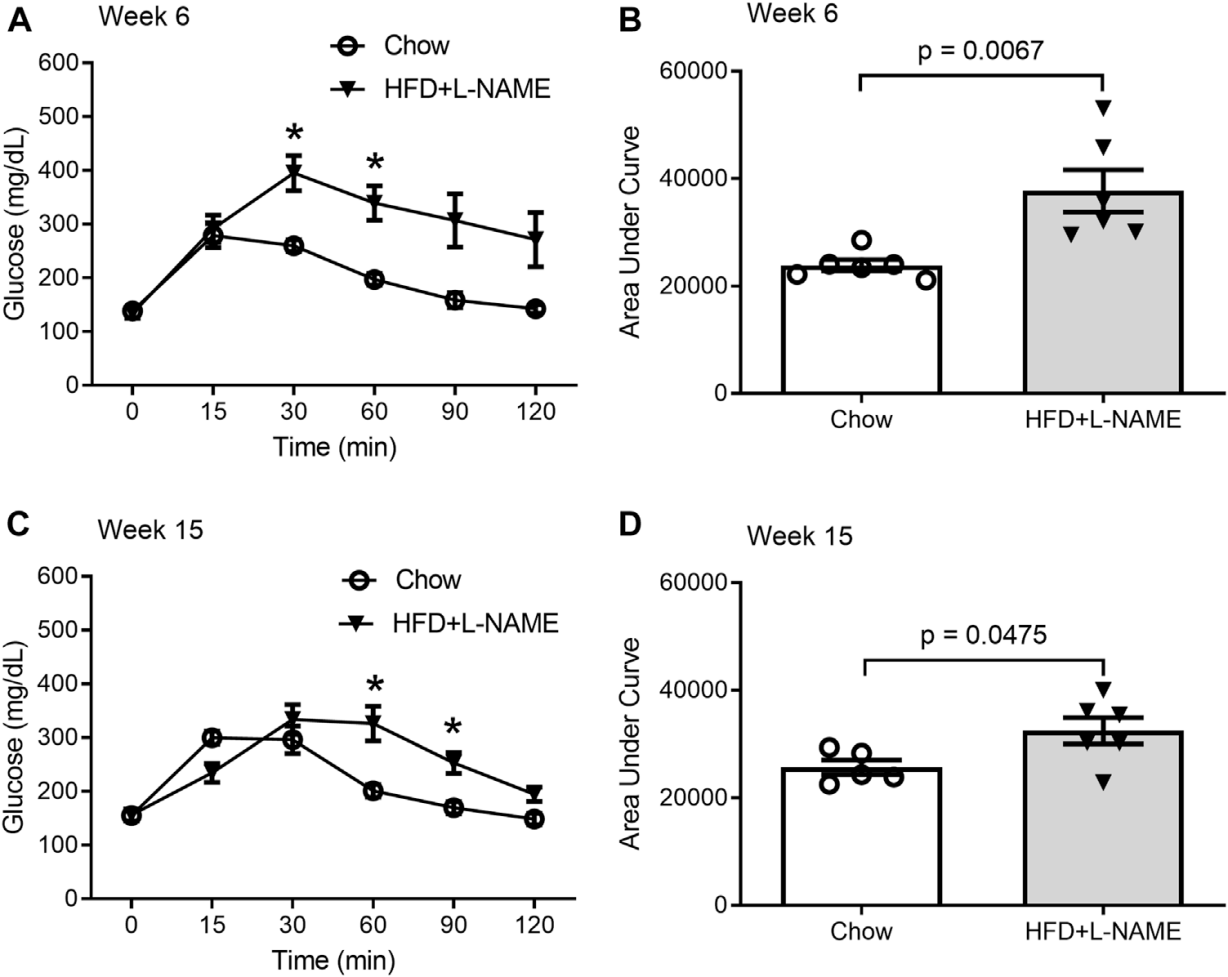
Glucose tolerance tests (GTTs) performed at week 6 and 15. A peritoneal injection of 20% glucose solution (1 g/kg) was administered after 6-h fasting. Blood from the tail was collected immediately before (0 min) and 15, 30, 60, 90, and 120 min after the injection for glucose concentration measurement. **(A,B)**, GTTs at week 6. Shown are the time course of blood glucose levels during GTTs **(A)** and the area under curve (AUC) comparison **(B)** between the Chow and the HFD+L-NAME groups. Multiple t-tests reveal the P values for comparisons at 0, 15, 30, 60, 90, and 120 min are respectively 0.791, 0.711, 0.003, 0.002, 0.016, and 0.031; * denotes that the difference at 30- and 60-min is statistically significant after correction for multiple comparisons using the Holm-Sidak method. **(C,D)**, GTTs at week 15 weeks. Shown are the time course of blood glucose levels during GTTs **(C)** and the area under curve (AUC) comparison **(D)** between the Chow and the HFD+L-NAME groups. Multiple t-tests reveal the P values for comparisons at 0, 15, 30, 60, 90, and 120 min are respectively 0.955, 0.017, 0.35, 0.008, 0.006, and 0.026; * denotes that the difference at 60 and 90 min is statistically significant after correction for multiple comparisons using the Holm-Sidak method. Each circle or dot in B and D represents a mouse and *p* values are derived from two-sided unpaired *t*-tests, the same for all subsequent Figures.

**FIGURE 3:**
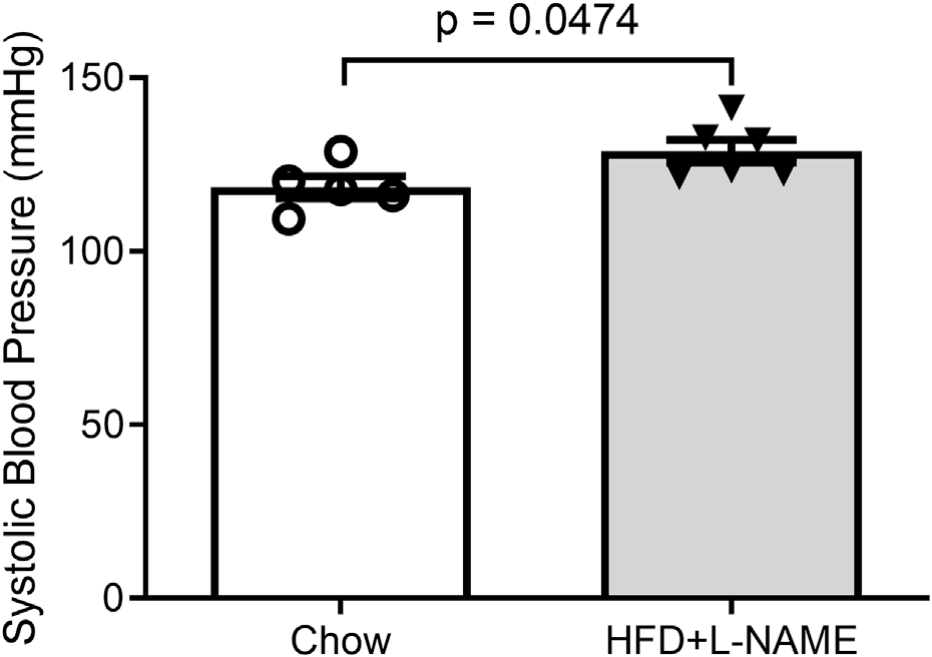
Systolic blood pressure measured at week 17 with the tail-cuff method.

Obesity can cause glucose intolerance; hence, we performed glucose tolerance tests on these animals at weeks 6 and 15. A bolus intraperitoneal injection of glucose induced increases in blood glucose levels in both groups, but the peak increase was significantly greater (week 6) and the increases lasted longer (weeks 6 and 15) in the HFD+L-NAME group compared with the Chow group (Figure 2), indicating that glucose intolerance was induced as early as week 6 by HFD+L-NAME. Administration of L-NAME is known to induce hypertension (Schiattarella et al., 2019); hence, we measured blood pressure using the tail-cuff method at week 17 and found that systolic blood pressure was significantly higher in the HFD+L-NAME group than in the Chow group (*p* = 0.0474, Figure 3). All three components of metabolic syndrome: hypertension, obesity, and glucose intolerance, are at least common comorbidities of HFpEF (Dunlay et al., 2017), and even can be an aspect of the multifactorial comorbidity driven pathology of the HFpEF syndrome (Tourki and Halade, 2021). The increased body weight, glucose intolerance, and hypertension observed in the HFD+L-NAME group are in alignment with recapitulating the metabolic syndrome central to HFpEF. and provide a catalyst for afterload induced cardiac remodeling of the left ventricle. Together, these pathologies formed the foundation for more advanced HFpEF manifestations.

### The “double-hit” method induced cardiac hypertrophy in FVB/N mice

**FIGURE 4:**
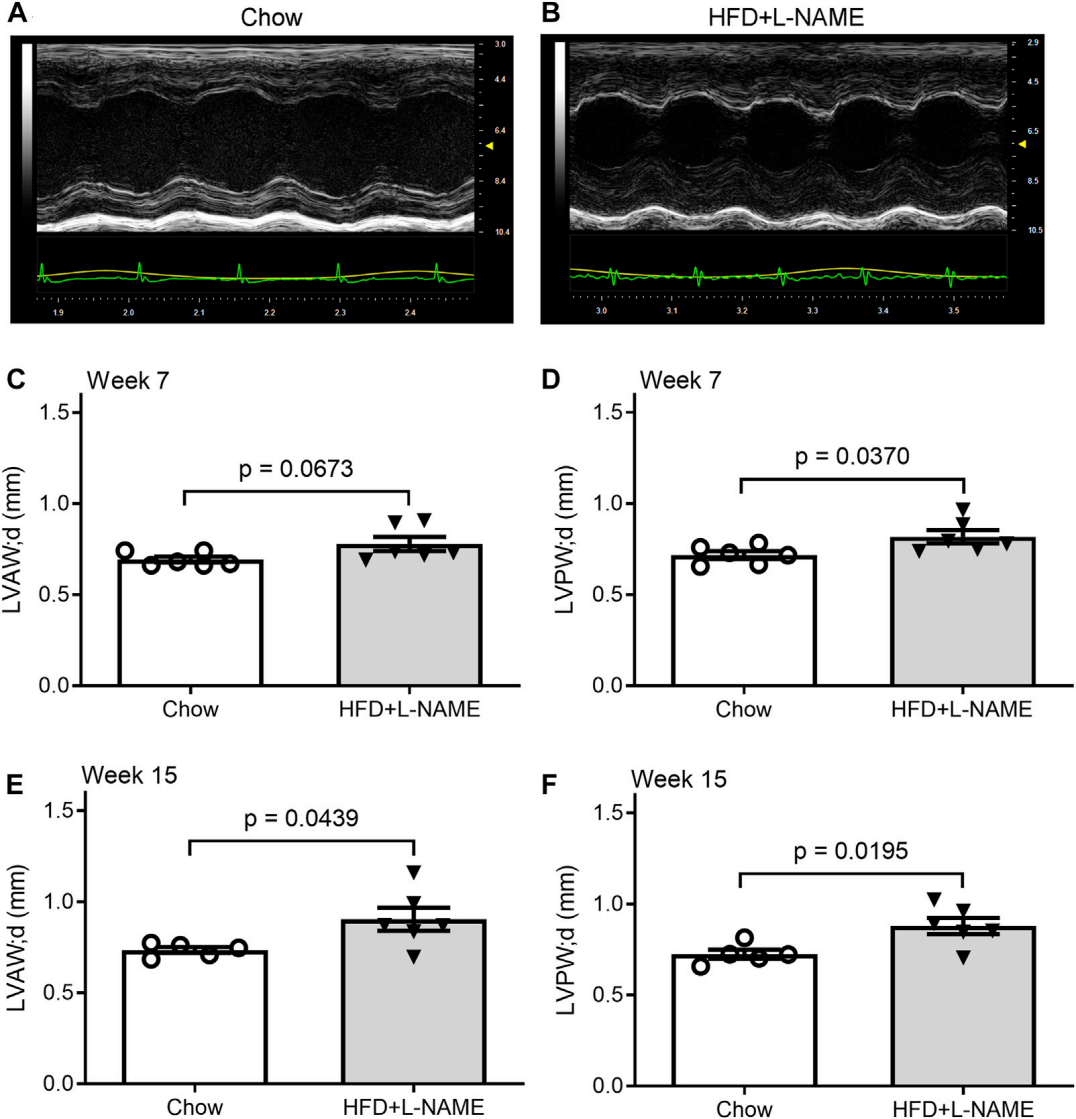
HFD+L-NAME induced cardiac hypertrophy as revealed by serial echocardiography. **(A,B)**, Representative echographs of the Chow and the HFD+L-NAME groups recorded at week 15. **(C)**, Left ventricular anterior wall thickness at the end of diastole (LVAW; d) at week 7. **(D)**, Left ventricular posterior wall thickness at the end of diastole (LVPW; d) at week 7. **(E)**, LVAW; d at week 15. **(F)**, LVPW; d at week 15.

**FIGURE 5:**
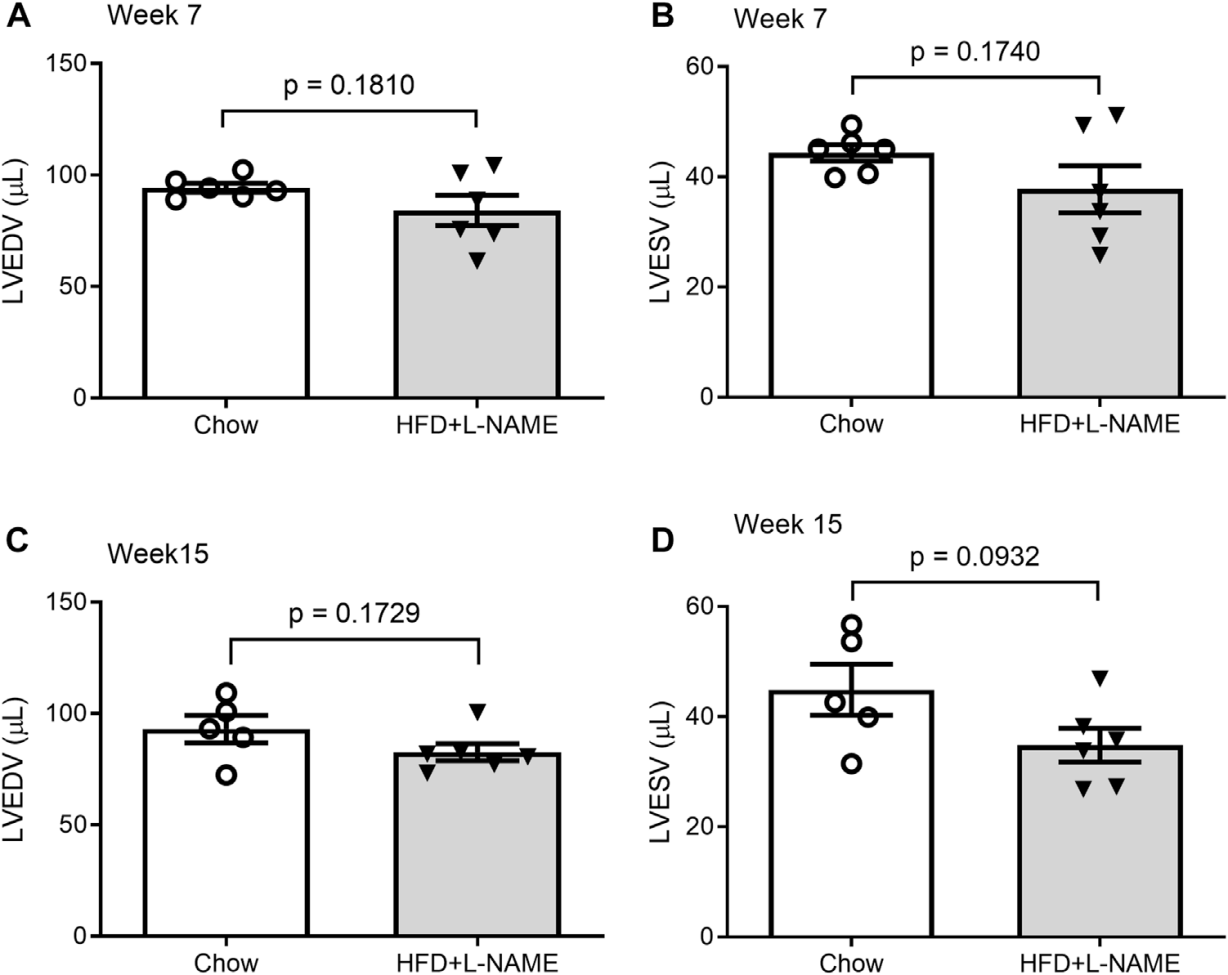
Comparisons of LV end-diastolic volume (LVEDV) and end-systolic volume (LVESV) between the Chow and the HFD+L-NMAE groups at week 7 **(A,B)** and week 15 **(C,D)**.

Serial echocardiography was performed to evaluate the dynamic changes in cardiac morphometry and function in live mice. Consistent with an increase in systolic blood pressure observed in the HFD+L-NAME group, left ventricular (LV) concentric hypertrophy was detected at both week 7 and week 15. This is evidenced by significantly greater LV end-diastolic posterior wall thickness (LVPW; d, Figure 4) and a tendency of deceased LV end-diastolic chamber diameter (Figure 5) in the HFD+L-NAME mice, compared with the Chow group disclosing diastolic dysfunction.

**FIGURE 6:**
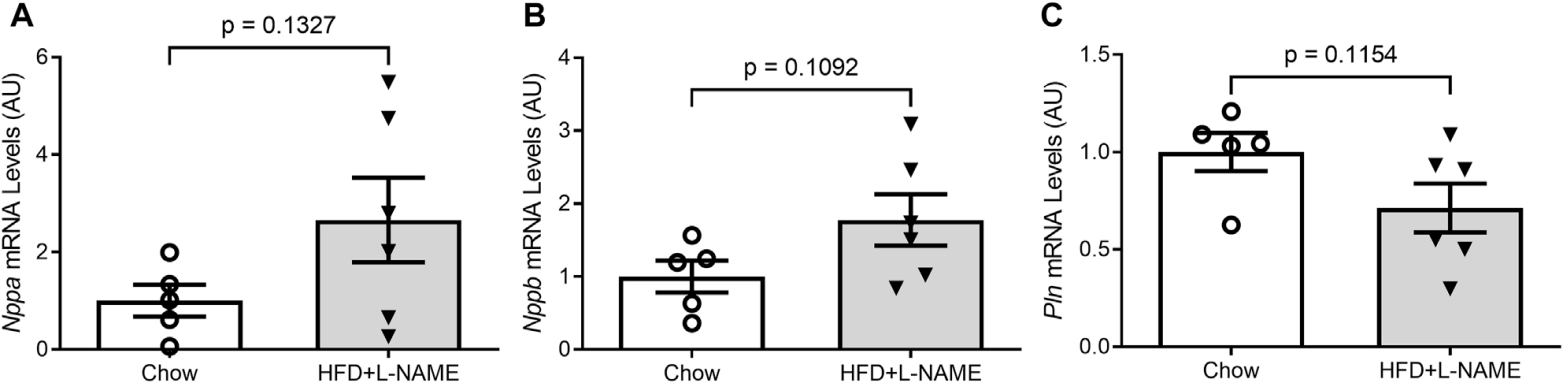
Reactivation of the fetal gene program in the ventricles. At the terminal experiment (week 18), LV myocardium was sampled, preserved in the RNA-Later reagent, and stored in −80°C until total RNA extraction. Total RNA was used for reverse transcription to synthesize the first-strand cDNA that was subsequently used for real time PCR to measure the mRNA levels with gene-specific primer sets for *Nppa* **(A)**, *Nppb* **(B)**, and *Pln* **(C)**.

At the terminal experiment, we detected that LV myocardial mRNA levels of fetal genes *Nppb* (*p* = 0.1092) and *Nppa* (*p* = 0.1327) tended to be higher and *Pln* mRNA levels (*p* = 0.1154) lower in the HFD+L-NAME group than in the Chow group (Figure 6) although the differences did not reach a statistical significance due to a large variation within each group.

### HFD+L-NAME impaired exercise tolerance in FVB/N mice

**FIGURE 7:**
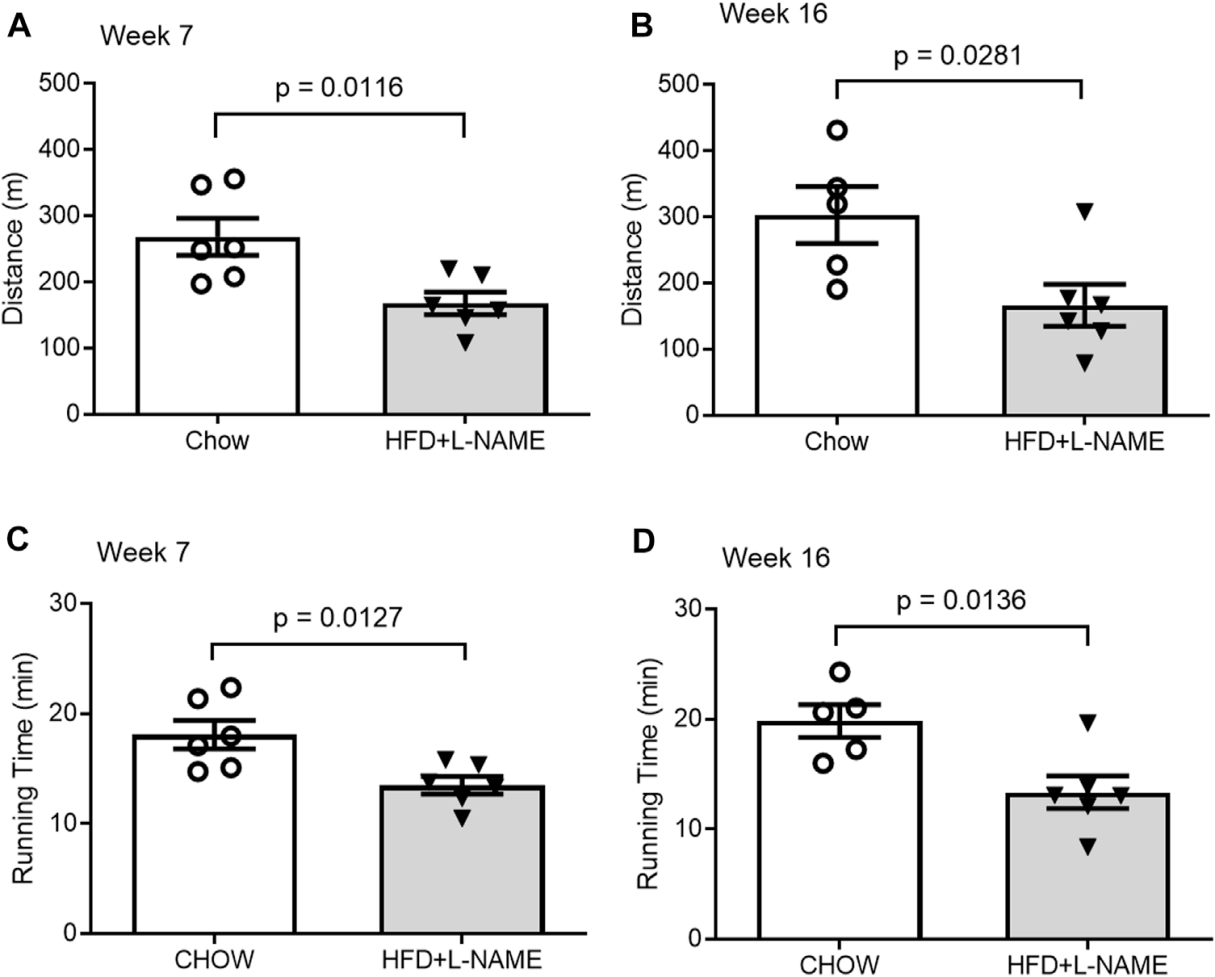
Comparisons of exercise tolerance between the Chow and the HFD+L-NAME groups at week 7 **(A,C)** and week 16 **(B,D)**.

Exercise tolerance tests performed at both weeks 7 and 16 revealed that the maximum distance and time of running to exhaustion by the HFD+L-NAME mice were markedly shorter than that by the Chow control group (Figure 7). At week 7 the Chow group ran an average of 269 m (18:07 min) while the HFD+L-NAME group ran an average of 168 m (13:30 min) prior to exhaustion. Similarly, at week 16 the Chow group ran 303 m (19:51 min) while the HFD+L-NAME group ran 167 m (13:21 min). These data indicate that exercise intolerance occurred as early as 7 weeks after HFD+L-NAME treatment. Reduced exercise tolerance including dyspnea and fatigue on mild exertion are common complaints seen in HFpEF patients clinically. The exercise intolerance in the HFD+L-NAME group indicate that the mouse model began to recapitulate the clinical aspects of HFpEF as early as week 7.

### Preserved ejection fraction

**FIGURE 8:**
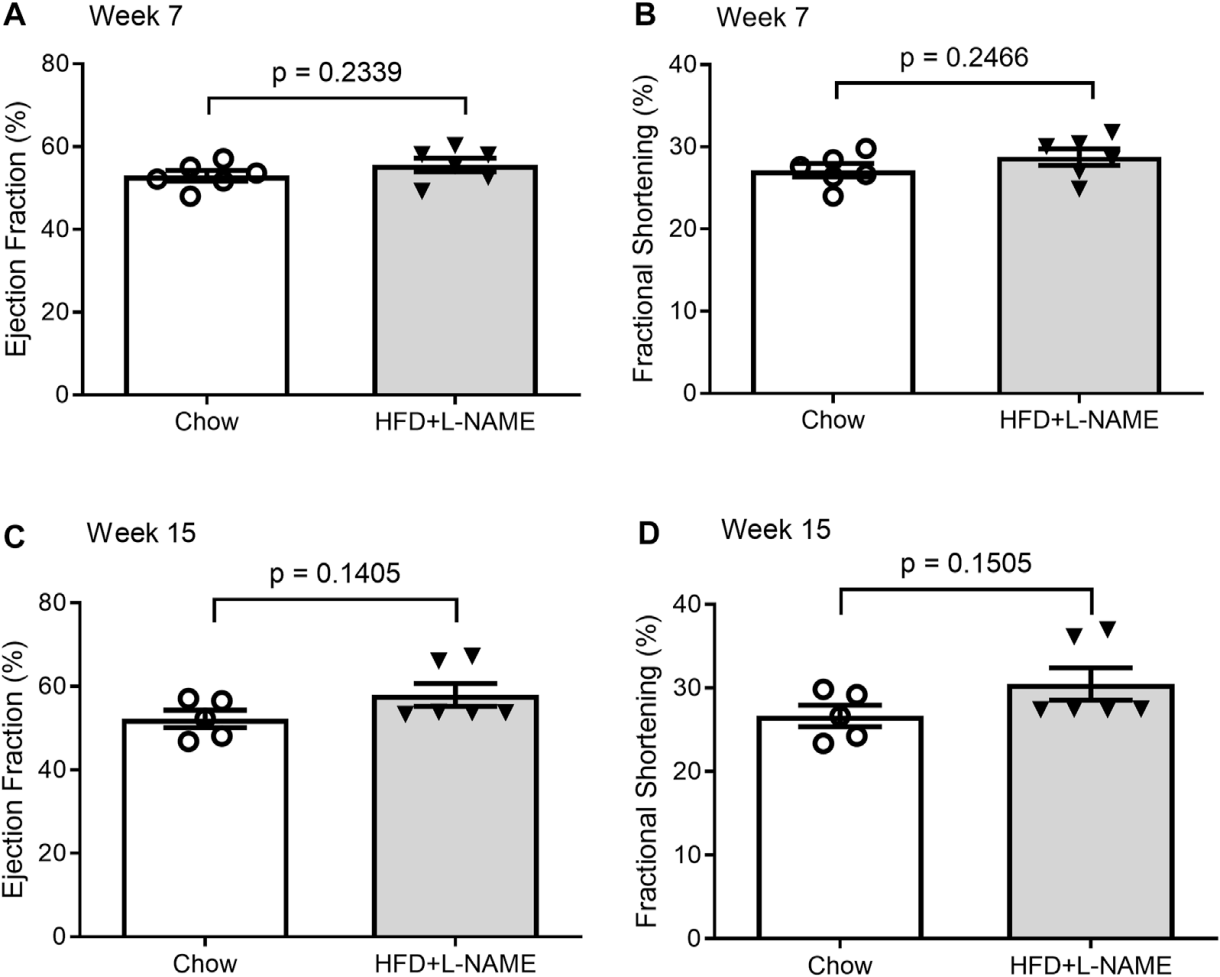
Comparisons of LV ejection fraction (EF) and fractional shortening (FS) between the Chow and the HFD+L-NMAE groups at week 7 **(A,B)** and week 15 **(C, D)**.

Despite the numerous characteristics of heart failure in the HFD+L-NAME treated mice, their LV ejection fraction (EF) and fractional shortening (FS) were not decreased throughout the study compared with those of the Chow control group (Figure 8), indicating that the systolic function was preserved in the HFD+L-NAME treatment group, one of the essential criteria for the diagnosis of HFpEF.

### Evidence of UPS impairment

**FIGURE 9:**
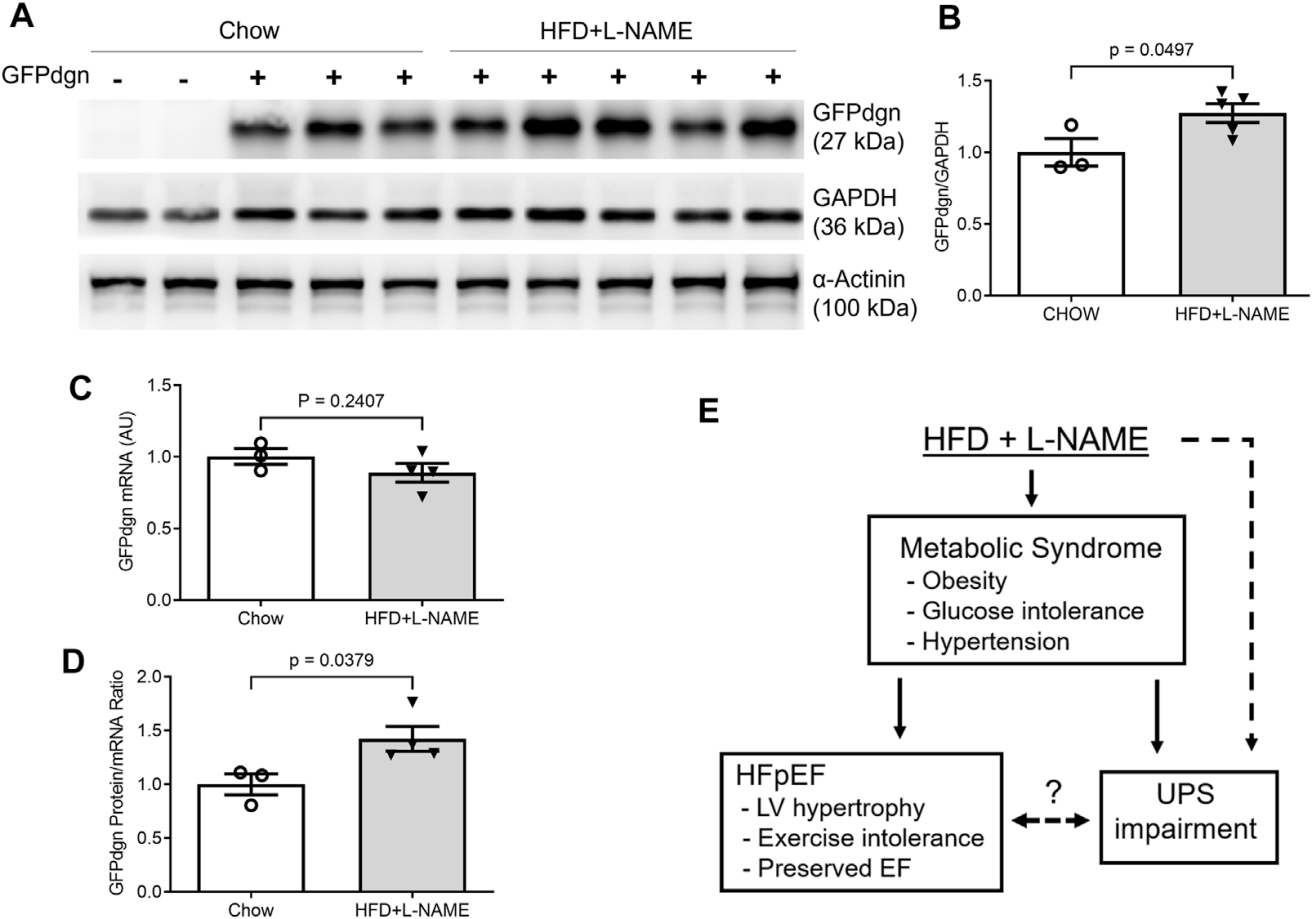
Myocardial mRNA and protein expression of GFPdgn in HFpEF and control mice. At the terminal experiment (week 18), a segment of left ventricular myocardium of each mouse was collected, snap-frozen in liquid nitrogen, and stored in a −80°C freezer until total protein extraction. The tissue preservation for total RNA extraction was the same as described in Figure 6. **(A,B)**, Representative images **(A)** and pooled densitometry data **(B)** of western blot analysis for myocardial GFPdgn. **(C)**, Comparison of GFPdgn mRNA levels as measured with qPCR. **(D)**, Comparison of GFPdgn protein/mRNA level ratios. **(E)**, A summary of overall findings. Dot line denotes a potential causative relationship. The question mark denotes the relationship remains to be determined. EF, ejection fraction; UPS, ubiquitin-proteasome system.

The GFPdgn transgenic mice, which were created and have been maintained in the FVB/N inbred background, have been extensively used to probe changes in UPS functioning in mice (Chen et al., 2005; Liu et al., 2006; Li et al., 2011); in absence of GFPdgn protein synthesis, the steady state level of GFPdgn protein inversely reflect UPS performance in the tissue or cell examined (Bence et al., 2001; Kumarapeli et al., 2005). We observed equivalent myocardial mRNA expression (*p* = 0.241) but significantly elevated GFPdgn protein levels (*p* = 0.0497) and greater GFPdgn protein to mRNA ratios (*p* = 0.0379) in the HFD+L-NAME treatment group compared with the CHOW group (Figure 9), indicating that elevated GFPdgn protein is not due to increased transcription, which provides compelling evidence that myocardial UPS performance is impaired in the HFpEF mouse.

## Discussion

HFpEF is one of the most debilitating, lethal, and prevalent healthcare challenges of the twenty-first century (Dunlay et al., 2017), with very limited proven treatments. The uncontrolled nature of HFpEF is largely due to the challenges in effectively recapitulating the disease in animal models and poorly understood of underlying pathogenic mechanisms (Horgan et al., 2014). The groundbreaking “two-hit” mouse model produced HFpEF in male C57BL/6N mice (Schiattarella et al., 2019), representing a milestone for HFpEF research. Here, we report successful emulation of HFpEF using the “two-hit” method in FVB/N mice, paving the way for use of mice with the FVB/N inbred background to further study HFpEF. This is important because many genetically engineered mouse models, especially those harboring a transgene driven by the murine Myh6 promoter, have been made in the FVB/N inbred background. The use of those mouse genetic models is expected to facilitate the experimental investigations into the pathogenesis and therapies of HFpEF. Moreover, taking advantage of the UPS performance reporter mouse, the present study also provides exciting evidence indicative of UPS malfunction in this HFpEF model, identifying a potentially new pathogenic pathway for HFpEF.

### Induction of HFpEF by the two-hit protocol in FVB/N male mice

The reactivation of the fetal gene program and a downregulation of *PLN* are often associated with and indicative of pathological hypertrophy and heart failure (Loffredo et al., 2014) (Man et al., 2018). Hence, these changes in gene expression are consistent with the significantly increased wall thickness revealed by echocardiography in the HFD+L-NAME group and the conclusion of induced cardiac remodeling in the HFD+L-NAME group. Clinically, diastolic dysfunction in HFpEF results in reduced cardiac output with maintained EF due to limited cardiac filling but at higher left ventricular end diastolic pressures. The increased systolic and diastolic wall thickness, while not essential, are characteristic of HFpEF as traditionally defined (Shah, 2013), while the reduction in *PLN* mRNA is indicative of the signature diastolic dysfunction of HFpEF (Grote Beverborg et al., 2021). Further, under the same “two-hit” hypothesis, diastolic dysfunction has been well described by invasively and non-invasively measured elevated LV filling pressures, histologically reported cardiac fibrosis and capillary rarefaction, pulmonary congestion, and reduction in coronary flow reserve. Although clear exercise intolerance was observed, the “two-hit” hypothesis is known to produce exercise intolerance without histopathological, molecular, or strength abnormalities in skeletal muscle indicating that decrement in exercise intolerance is not due to a skeletal muscle deficiency (Schiattarella et al., 2019). Taken together, the hypertensive afterload stress and metabolic syndrome pathologies, likely created an environment for pathologic hypertrophic cardiac remodeling and diastolic dysfunction to occur, a hallmark of HFpEF. Despite these changes, serial echocardiographic analyses displayed preserved ejection fraction across both groups through at least week 15 (Figure 8). Therefore, the present study demonstrates compellingly that HFpEF can be produced with the “two-hit” protocol in male FVB/N mice.

HFpEF is traditionally considered a disease of the elderly resulting in challenges studying the disease *in vivo* in a time and cost-efficient manner. To construct a model that most quickly emulates the complete severity and mortality of HFpEF, male mice were used exclusively (Patten, 2007) (Tromp et al., 2018). In an aim of striking a balance between time efficiency and model validity, mice six to 7 months of age were used. While the maiden voyage of the two-hit hypothesis successfully recapitulated the disease using mice 8–12 weeks of age (Schiattarella et al., 2019), mice 3 months senior likely accounted for the age-related etiology of HFpEF to a greater degree.

Conventionally, the “two-hit” HFpEF mouse model uses C57BL/6N wild-type mice (Schiattarella et al., 2019), a lineage traditionally renowned as the gold standard for producing diet induced obesity and metabolic syndrome. However, recent studies comparing FVB/N and C57BL/6J have begun to question this notion. FVB/N ob/ob and C57BL/6J ob/ob mice are used to study obesity and its comorbidities as both murine lineages are leptin deficient thus maximizing hunger and the effects of the HFD. Compared to the C57BL/6N ob/ob lineage, FVB/N ob/ob mice demonstrate increased hyperglycemia, whole body and muscle insulin resistance, and reduced clearance of circulating triglycerides (Haluzik et al., 2004), suggesting that the FVB/N murine lineage exhibits significant benefits over the C57BL/6J strain in modeling metabolic dysfunction, a quintessential comorbidity of HFpEF. Likewise, Nascimento-Sales et al. (2017), found that while C57BL/6N mice develop obesity more quickly than FVB/N mice, FVB/N mice actually show a significantly greater degree of metabolic intolerance after treatment with a HFD as evidenced by higher insulin resistance, greater liver steatosis, and a large degree of epididymal white adipose tissue induced inflammation. Notably, they conclude by recommending the FVB/N lineage as a new tool to uncover the complex multifactorial symptoms of obesity and metabolic syndrome of which HFpEF is inevitably intertwined.

Between 40%–50% of HFpEF patients are obese (Shah et al., 2013a). Similarly, 45% of HFpEF patients have diabetes (McHugh et al., 2019). However, due to the decreased prevalence of diabetes relative to obesity, HFpEF is more specific towards the diabetic population. This is perhaps because HF and metabolic syndrome share numerous underlying derangements that result in increased HF risk including insulin resistance, metabolic derangements, endothelial dysfunction, oxidative stress, mitochondrial dysfunction, and autonomic neuropathy (Dhingra and Vasan, 2012; Oktay et al., 2013). Type 2 diabetes mellitus (T2DM) shares the pathologies of sodium retention, impaired skeletal muscle function, and metabolic derangements with HFpEF, implying an integrated pathogenesis of both diseases, (Shah et al., 2013b). As more insight into comorbid HFpEF and T2DM is acquired, it is anticipated physicians will be better equipped to provide effective unique treatments for commonly comorbid HFpEF and T2DM.

Further, Reduced exercise tolerance and increased myocardial remodeling are critical features of HFpEF (Paulus and Tschope, 2013; Gevaert et al., 2019). FVB/N mice demonstrate higher capabilities and cooperativity of daytime exercise tolerance testing (Gibb et al., 2016). In response to exercise tolerance training, FVB/N mice display increased cardiac remodeling compared to C57BL/6J mice (Massett and Berk, 2005; Gibb et al., 2016). A higher level of myocardium hypertrophy in response to increased exercise stress is suggestive that FVB/N mice may also develop a greater degree of cardiac remodeling due to increased afterload stress elicited by HFpEF hypertension.

Mouse models emulating HFpEF generally originate from three different avenues of the most prominent risk factors of HFpEF: hypertensive models, diabetic (obesity) models, and aging models (Horgan et al., 2014). The novel “two-hit” hypothesis developed a HFpEF mouse model from both the hypertensive and diabetic avenues. The FVB/NJ mice strain has been used to illuminate the pathologies of HFpEF through the aging model avenue (Koch et al., 2013). We report a HFpEF mouse model that potentially utilizes all three avenues of the most significant HFpEF murine models (hypertensive, metabolic dysfunction, and aging) to emulate HFpEF. Finally, applying the “two-hit” protocol to the FVB/NJ mice may not only emulate the clinical symptoms of HFpEF but could also optimize the exercise tolerance measurements, essential cardiac remodeling, and circadian misalignments of the disease (Massett and Berk, 2005; Gibb et al., 2016). A deeper understanding of the “two-hit” hypothesis and HFpEF pathophysiology may be explored by expanding the protocol across murine lineages, namely, the FVB/NJ lineage.

### Impairment of UPS performance in HFpEF mice

The UPS is the primary pathway responsible for the targeted degradation of abnormal, misfolded, damaged, or oxidized proteins, in addition to for the degradation of normal but no longer needed proteins (Wang et al., 2013). In response to the recent reports of accumulated misfolded proteins (Gonzalez-Lopez et al., 2015) and altered unfolded protein response (UPR) playing a pivotal role in the pathogenesis of HFpEF (Schiattarella et al., 2019), we hypothesized that myocardial UPS performance could be impaired during HFpEF. Beginning to examine this hypothesis, the present study applied the “two-hit” protocol to the transgenic mice with ubiquitous expression of GFPdgn, a surrogate substrate of the UPS (Kumarapeli et al., 2005). In absence of changes in GFPdgn protein synthesis, changes in its steady-state protein levels reflect inversely UPS performance (Kumarapeli et al., 2005). Notably, the GFPdgn mRNA expression between CHOW and HFD+L-NAME groups was not significantly different, but GFPdgn protein levels were elevated in the HFD+L-NAME group (Figure 9), indicating diminished UPS function in HFpEF. Such impairment is consistent with the other preliminary studies suggesting that the accumulation and mismanagement of misfolded, damaged, and abnormal proteins play a vital role in the development of HFpEF (Wang et al., 2014; Gonzalez-Lopez et al., 2015; Schiattarella et al., 2019).

Evidence of impaired UPS functioning in HFpEF beckons for further investigation to provide a deeper understanding of the pathogenesis and devise new treatment strategies for HFpEF. For example, it will be important to elucidate the main factors that impair UPS performance during HFpEF. To this end, both diabetes and hypertension have been shown to impair myocardial UPS performance and proteasome functional insufficiency (Ranek et al., 2015; Li et al., 2017), but more detailed molecular mechanisms remain to be delineated. It is also conceivable that UPS impairment could affect myocardial proteostasis and thereby cardiac function through both slowing down the turnover of normal proteins and accumulating abnormal proteins, and both are quite detrimental to the heart in many ways. Ultimately, we report successful modeling of HFpEF in male FVB/N mice and, by taking advantage of a transgenic UPS reporter mouse, we have detected myocardial UPS functioning impairment during HFpEF, suggesting a pathogenic role for impaired protein degradation in the development and progression of HFpEF but insist on further exploration (Figure 9E).

### Limitations and future directions

This study has potential limitations. While the power of the study was sufficient for the primary readouts, the number of mice per group is not enough to decipher whether the GFPdgn transgenic expression has an impact on the occurrence of HFpEF. No female mice were included, leaving it untested whether the “double hit” method could cause HFpEF in female FVB/N mice or not. Additionally, mice were treated with HFD+L-NAME for up to 18 weeks. Despite both Schiattarella et al. and our results providing convincing evidence of HFpEF within this time frame, the pathogenesis of HFpEF is known to develop over the course of decades in humans (Lam et al., 2011). It begs the question, was HFpEF established in its completeness within the 18-week treatment course? Would longer treatment periods elicit models with grander disease severity, more confluent outcomes, and ultimately a clearer window into HFpEF etiologies? In response, the degree of HFpEF could be graded through measurement of the E/A ratio during echocardiology. Murine blood pressures were measured using tail-cuff methodology instead of telemetry. While tail-cuff methods have been validated against telemetry for measuring more course changes in blood pressure, telemetry allows for a longer period of measurement, giving greater power to the study so that fewer animals are needed (Fraser et al., 2001). Likewise, echocardiology was used to measure ventricular wall thickness as a surrogate for cardiomyocyte hypertrophy, fibrosis, and increasing filling pressures when histological data using wheat germ agglutinin, hematoxylin and eosin staining, and pressure volume loop data would have measured these more directly. Analysis of *Nppa, Nppb, Pln,* and *GFPdgn* may have been more suited for normalization with *36B4* rather than *GAPDH* as *GAPDH* has been shown to change during obesity and metabolic syndrome (Fan et al., 2020). Despite preliminary affirmation of the role of UPS malfunction in the pathogenesis of HFpEF, more in-depth deciphering the specific defect in UPS-mediated protein degradation pathway will provide concise guidance for therapeutic interventions should the UPS impairment play an important pathogenic role. It will be extremely important to address questions such as “does HFpEF drive UPS dysfunction or does UPS dysfunction drive HFpEF?”

In summary, the present study demonstrates for the first time that impaired UPS performance can occur in an animal model of HFpEF; thus, further investigation into a potential cause-effect relationship between UPS impairment and HFpEF is warranted.

## Data Availability

The original contributions presented in the study are included in the article/supplementary materials, further inquiries can be directed to the corresponding author.
