# Supplementary figures and images for: The double-hit protocol induces HFpEF and impairs myocardial ubiquitin-proteasome system performance in FVB/N mice
Source: Front Physiol. 2023 Jun 8;14:1208153. doi: 10.3389/fphys.2023.1208153 (PMC10285383; doi:10.3389/fphys.2023.1208153)

Uncropped gel images of Figure 9

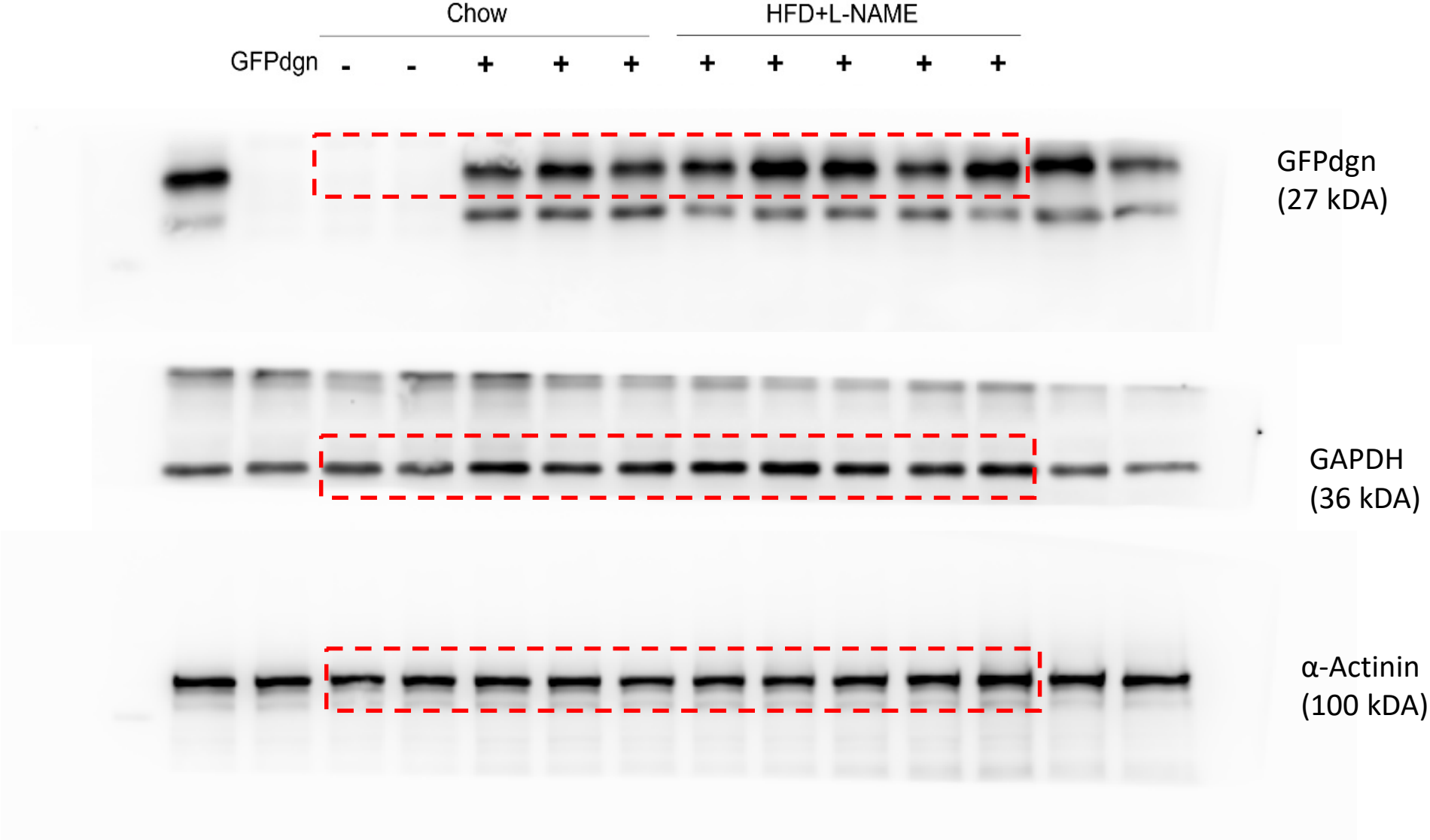

Supplement: Supplementary file 1 [file DataSheet1.PDF]
